# Graz Endocrine Causes of Hypertension (GECOH) study: a diagnostic accuracy study of aldosterone to active renin ratio in screening for primary aldosteronism

**DOI:** 10.1186/1472-6823-9-11

**Published:** 2009-04-07

**Authors:** Stefan Pilz, Andreas Tomaschitz, Vinzenz Stepan, Barbara Obermayer-Pietsch, Astrid Fahrleitner-Pammer, Natascha Schweighofer, Horst R Portugaller, Harald Sourij, Harald Dobnig, Andreas Meinitzer, Thomas R Pieber

**Affiliations:** 1 Department of Internal Medicine, Division of Endocrinology and Nuclear Medicine, Medical University of Graz, Auenbruggerplatz 15, 8036 Graz, Austria; 2 Department of Radiology, Medical University of Graz, Auenbruggerplatz 9, 8036 Graz, Austria; 3 Clinical Institute of Medical and Chemical Laboratory Diagnostics, Medical University of Graz, Auenbruggerplatz 15, 8036 Graz, Austria

## Abstract

**Background:**

Primary aldosteronism (PA) affects approximately 5 to 10% of all patients with arterial hypertension and is associated with an excess rate of cardiovascular complications that can be significantly reduced by a targeted treatment. There exists a general consensus that the aldosterone to renin ratio should be used as a screening tool but valid data about the accuracy of the aldosterone to renin ratio in screening for PA are sparse. In the Graz endocrine causes of hypertension (GECOH) study we aim to prospectively evaluate diagnostic procedures for PA.

**Methods and design:**

In this single center, diagnostic accuracy study we will enrol 400 patients that are routinely referred to our tertiary care center for screening for endocrine hypertension. We will determine the aldosterone to active renin ratio (AARR) as a screening test. In addition, all study participants will have a second determination of the AARR and will undergo a saline infusion test (SIT) as a confirmatory test. PA will be diagnosed in patients with at least one AARR of ≥ 5.7 ng/dL/ng/L (including an aldosterone concentration of ≥ 9 ng/dL) who have an aldosterone level of ≥ 10 ng/dL after the saline infusion test. As a primary outcome we will calculate the receiver operating characteristic curve of the AARR in diagnosing PA. Secondary outcomes include the test characteristics of the saline infusion test involving a comparison with 24 hours urine aldosterone levels and the accuracy of the aldosterone to renin activity ratio in diagnosing PA. In addition we will evaluate whether the use of beta-blockers significantly alters the accuracy of the AARR and we will validate our laboratory methods for aldosterone and renin.

**Conclusion:**

Screening for PA with subsequent targeted treatment is of great potential benefit for hypertensive patients. In the GECOH study we will evaluate a standardised procedure for screening and diagnosing of this disease.

## Background

Primary aldosteronism (PA) is characterised by aldosterone concentrations that are inappropriately high in relation to the activity of the renin-angiotensin system and that are not adequately suppressible by sodium loading [1-3]. Recent studies have shown that PA, with its two main subtypes aldosterone producing adenoma (APA) and bilateral adrenal hyperplasia (BAH), is the most common cause of secondary hypertension with a prevalence of approximately 5–10% among all hypertensive patients and an even higher prevalence among selected patients with advanced stages of hypertension and resistant hypertension [1-3]. Importantly, patients suffering from PA have, independent of blood pressure, an increased risk of cardiovascular diseases and renal damage when compared to patients with essential hypertension [4-7]. Therefore, screening for PA is important for hypertensive patients because detection of PA offers the opportunity for a targeted and effective treatment that significantly reduces the excess cardiovascular risk in patients with PA [7,8]. After removal of the affected adrenal gland, patients with APA are cured from hypertension in approximately half of the cases with an improvement of hypertension in the remainder [1-3]. This surgical therapy is also considered cost-effective [9]. Patients with BAH respond well to drug therapy with mineralcorticoid receptor antagonists [1-3,7].

Despite a wide acceptance of the aldosterone to active renin ratio (AARR) and the aldosterone to renin activity ratio (ARR) in screening for PA, the accuracy of these tests is still not well documented [10], leading to many controversies and discrepancies in the use of AARR and ARR as a screening tool for PA which can be attributed to several causes: (1) There exists no generally accepted "gold standard" or "reference standard" for the diagnosis of PA, mainly because APA but not BAH can be unambiguously diagnosed (APA can be diagnosed by cure or improvement of arterial hypertension after surgical removal of an histopathologically confirmed adrenal adenoma). (2) In most diagnostic studies in this field only patients with a positive result of the screening test were referred to the chosen "reference standard test", which may be responsible for a verification bias with an overestimated accuracy of the screening test[10]. (3) The results of the AARR and/or ARR are hardly comparable between different laboratories. Comparative studies have partially shown significantly different inter-laboratory results, mainly depending on the laboratory methods (assays) used [1-3,11]. (4) The AARR and/or the ARR are also influenced by several other factors including e.g. medications, age, time of blood sampling, posture and dietary salt intake. It still needs to be clarified whether these above mentioned factors, in particular the use of beta-blockers, that were shown to decrease renin levels, significantly alter the accuracy of the AARR and/or ARR in screening for PA [1-3,12,13]. It should also be pointed out that dietary salt loading has a significant impact on the renin-angiotensin aldosterone system (RAAS) with complex interactions and is thus a potential confounder for diagnostic procedures for PA [14], which are largely performed without considering dietary sodium intake [1]. In summary, previous studies reveal great discrepancies concerning the blood sampling conditions and laboratory methods for the determination of the AARR and/or ARR. Hence there is an urgent need for further evaluation and development of standardised and practicable diagnostic procedures for the detection of PA.

In our study we apply a standardised diagnostic procedure for PA in hypertensive patients that was derived from the most valid currently available data about this topic [1-3]. Our main study aims are (1) to test the accuracy of the AARR in screening for PA (primary outcome), (2) to test the accuracy of the saline infusion test (SIT) and (3) to evaluate whether the use of beta-blockers significantly alters the accuracy of the AARR as a screening test for PA. (4) In the first 100 patients of our study we aim to compare our laboratory methods for aldosterone and renin with other widely used and validated assays and (5) we will calculate the accuracy of the ARR in diagnosing PA and compare it with the accuracy of the AARR. (6) We will also evaluate the test characteristics of the SIT in comparison with PA diagnosis based on 24 hours urine aldosterone levels.

## Methods and design

### Study design

**Figure 1:**
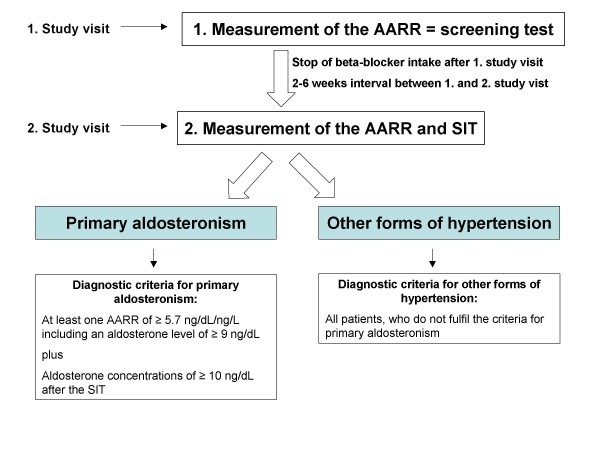
**Flow-chart of the GECOH study including diagnostic criteria for primary aldosteronism and other forms of hypertension**. See text for detailed information.

The Graz endocrine causes of hypertension (GECOH) study is a single center diagnostic accuracy study to determine the sensitivity and specificity of the AARR in screening for PA (Figure 1). The GECOH study was designed by adhering to the recommendations of the statement for reporting studies of diagnostic accuracy (STARD) and the Declaration of Helsinki [15].

### Study population and study setting

The study will be performed at an outpatient clinic of a tertiary care center (Department of Internal Medicine, Division of Endocrinology and Nuclear Medicine, Medical University of Graz, Austria). The study population consists of patients who are routinely referred to our department for screening for endocrine hypertension. The patient management and disease classification will be done by experienced endocrinologists.

### Inclusion/Exclusion criteria

Medications that significantly interfere with aldosterone mediated effects including spironolactone, canrenoate, eplerenone, amiloride and triamteren have to be stopped at least 4 weeks before screening procedures for the GECOH study will be carried out because it is generally recommended to stop these medications before testing for PA [1].

#### Inclusion criteria

The main inclusion criterion is arterial hypertension defined according to recent guidelines as an average office blood pressure on at least two occasions of systolic ≥ 140 and/or diastolic ≥ 90 mmHg or a mean 24 ambulatory blood pressure of systolic ≥ 125 and/or diastolic ≥ 80, or a home blood pressure of systolic ≥ 130 and/or diastolic ≥ 85 or ongoing antihypertensive treatment that was initiated due to arterial hypertension [16,17]. Further inclusion criteria are age of ≥ 18 years and written informed consent.

#### Exclusion criteria

Exclusion criteria were chosen to avoid inclusion of patients whose AARR is likely to be significantly biased by underlying diseases or medications. As a matter of course patients in whom the SIT may be contraindicated will be excluded too [1]. Accordingly, the exclusion criteria are a glomerular filtration rate (GFR) according to the MDRD formula < 30 ml/min/1.73 m^2 ^[18], severe hepatic failure (Child-Pugh class B or C), severe heart failure with NYHA class 3 or 4, acute coronary syndrome within the last two weeks, immunosuppressive therapy, glucocorticoid therapy, ongoing chemotherapy, pregnancy and any other disease with an estimated life expectancy below 1 year. Patients that are initial eligible for the GECOH study but who meet any of the exclusion criteria during the study will also be excluded.

### Blood sampling conditions

For all blood samplings within this study, each patient will be informed to come fasting (12 hour overnight fast). Smoking and intake of any antihypertensive drug should also be avoided in the morning before blood collection. Blood samplings for the determinations of the AARR and ARR will be done in the morning (8:00 to 11:00) after the patients have been seated for 10 minutes. Extreme care will be taken to avoid haemolysis and prolonged stasis.

### Screening test

The AARR (aldosterone in ng/dL divided by active renin concentration in ng/L) is used as the screening test for PA [1-3]. An elevated AARR indicates that the aldosterone secretion is inappropriately high with regard to its principle trophin renin [1-3]. The chosen cut-off value for a positive screening test of an AARR of ≥ 5.7 (corresponding to an ARR of 30 expressed as aldosterone in ng/dl divided by plasma renin activity [PRA] in ng/ml/h) is in line with recent guidelines (1). Furthermore, only patients with an aldosterone of ≥ 9 ng/dL, in addition to an AARR of ≥ 5.7, are considered to have a positive screening test because a recent study has shown that there were no patients with PA that had aldosterone levels below this threshold [19].

### Reference standard test

All patients will undergo the reference standard test that consists of two determinations of the AARR (including the AARR of the screening test) plus the results of the SIT. The rationale for using two measurements of the AARR derives from the observations of Tanabe et al who have shown that repeated determinations of the AARR are necessary to avoid overlooking of patients with PA [20]. In the GECOH study the second determination of the AARR will be done 2–6 weeks after the screening test and is immediately followed by the SIT on the same day.

The SIT is a safe, simple and relatively inexpensive confirmatory test for patients with a positive screening test [1,21]. The SIT is widely used and shows a good agreement with the fludrocortisone suppression test (FST), which is by some authors considered as the gold standard test for diagnosing PA [21]. However, the FST requires hospitalization for 4 days and intake of fludrocortisone tablets together with salt and potassium supplementation and is thus less practicable [21]. In the morning of the SIT blood will be drawn for the second determination of the AARR after seating for 10 minutes. Then the patient will stay in a recumbent position for one hour. Afterwards the SIT is performed by infusing 2 litres of 0.9% saline i.v. over 4 hours starting at 9:00–9:30 [1]. After the saline infusion, blood sampling will be performed and patients with an aldosterone concentration ≥ 10 ng/dL are considered to have a positive SIT. This cut-off value is in line with the recommendation of Diederich et al for our specific aldosterone assay [22] and is supported by previous results from Schirpenbach et al [11].

In addition, we will also measure 24 hours urine sodium and aldosterone levels in the morning before the SIT. This will be done because a high chronic oral sodium loading indicated by 24 hours urine sodium concentrations of over 200 mEq [23] is part of an established confirmatory test, in which the diagnosis of PA is based on 24 hours urine aldosterone concentration above 12 μg/24 h[1,24] Considering that according to recent guidelines we do not make any recommendations concerning salt intake of our study participants it is likely that a significant proportion of our study population will have a 24 hours urine sodium level above 200 mEq[23] In such patients we will compare the SIT with the results of the 24 hours urine aldosterone measurements in order to evaluate the accuracy of the SIT in the setting of high dietary sodium load.

### Use of medications in the GECOH study

Except of drugs that significantly interfere with aldosterone mediated effects and that must be withdrawn at least four weeks before inclusion into the GECOH study, the remainder medical therapy will not be altered for the screening test. This approach is supported by previous findings showing that concomitant antihypertensive medication does not adversely affect the test accuracy of the ARR in diagnosing PA [24]. However, this issue still remains to be addressed for the AARR and warrants confirmation in further studies because several medications have been shown to alter the aldosterone to renin ratio [1]. Beta-blockers are, apart from the above mentioned drugs that interfere with aldosterone mediated effects, the medications that are considered to have the greatest impact on the aldosterone to renin ratio[12,13] This effect of beta-blockers on the AARR is strongly driven by their effect on renin levels with minor effects on aldosterone concentrations [1]. To evaluate whether the use of beta-blockers significantly alters the accuracy of the AARR in screening for PA we will stop the intake of beta-blockers in all patients after the screening test in order to have a second AARR determination that is not potentially biased by beta-blocker intake and that can be compared with the screening test with regard to its sensitivity and specificity in detecting PA. In case of poor blood pressure control after cessation of beta-blockers we will either increase the dose of other pre-existing antihypertensive drugs or we will initiate a therapy with verapamil, hydralazine, prazosin, doxazosin and/or terazosin, that have only minor influences on the AARR [1].

### Diagnostic criteria

According to the results of the reference standard test we have established the following criteria for PA and for other forms of arterial hypertension:

Patients with at least one AARR ≥ 5.7 including an aldosterone levels ≥ 9 ng/dL who have aldosterone concentrations of ≥ 10 ng/dL after the SIT will be diagnosed as having PA. These criteria are in line with the general recommendation that the diagnosis of PA should always be based on a positive screening test (AARR) that must be confirmed by a confirmatory test (SIT) [1-3]. Patients who do not fulfil the above mentioned criteria for PA will be diagnosed as having other forms of arterial hypertension.

### Laboratory methods

All blood samples taken will be centrifuged within 1 hour after sampling and will be either immediately stored at -20°Celsius until analysis, that will be done on a weekly basis, or measured at least 4 hours after blood collection. Before analysis or freezing all samples will be kept at room temperature, except of the samples for determination of aldosterone, which will be kept at 4°Celsius.

Active renin concentrations will be determined according to manufacturers instructions from EDTA plasma by a "RENIN III GENERATION" (GEN. III) RIA assay (Renin IRMA RIA-4541, DRG Instruments GmbH, Marburg, Germany), that has been calibrated against a WHO standard [25]. The lower detection limit of this assay is < 1.0 ng/L and renin concentrations of 1.0 ng/L or lower are all set 1.0 ng/L. Intra-assay and inter-assay coefficients of variation of this assay are 0.6 to 4.5 and 2.7 to 14.5%, respectively. According to recent guidelines the conversion factor from a PRA level in ng/ml/h to renin in pg/ml is 5.2 and the conversion factor from PRA in ng/ml/h to renin in mU/L is 8.2 [1]. These latter conversion factors were recommended for a II generation RIA (Bio-Rad Renin II RIA) and these II generation RIAs are in excellent agreement with our III generation renin assay, so that we adopted these conversion factors [1,25].

Aldosterone from EDTA plasma will be determined according to manufacturers instructions by RIA (Active Aldosterone RIA DSL-8600, Diagnostic Systems Laboratories, Inc., Webster, Texas, USA) [11]. The intra-assay and inter-assay coefficients of variations of this assay are 3.3 to 4.5 and 5.9 to 9.8%, respectively.

Measurements of plasma renin activity, that are based on the determination of angiotensin I whose conversion from angiotensinogen is catalyzed by renin, will be performed in blood samples of the screening test of the first 100 patients included in the GECOH study. Renin activity in EDTA plasma will be measured by an angiotensin-I-RIA (RENCTK, Dia Sorin, Italy) according to manufacturers' instructions. In the same subset of patients (first 100 patients of this study) we will also determine aldosterone levels in serum of the screening test by liquid chromatography – tandem mass spectrometry according to the method by Turpeinen et al. [26]. Measurements of 24 hours urine aldosterone levels will also be done by means of liquid chromatography – tandem mass spectrometry [27]. As part of the clinical routine, additional laboratory measurements for other causes of arterial hypertension (e.g. for pheochromocytoma or Cushing's disease) and cardiovascular risk assessments may also be performed in patients of the GECOH study. All laboratory measurements will be done at the laboratory of the Medical University of Graz, Austria.

### Sample size

Sample size calculation was carried out for our primary outcome (accuracy of the AARR in screening for PA) according to the previously published method by Jones et al [28]. This method is used to calculate the sample size required to estimate an expected level of sensitivity with a predefined degree of precision (confidence interval) [28]. According to previous results of hypertensive patients referred to tertiary care centres we estimated a prevalence of PA of 20% among our study population [1-3,29,30]. Using this disease prevalence of 20% and an expected level of sensitivity of the AARR in screening for PA of 95% [3,22] with a confidence interval of 5% we calculated a required study sample size of 365 study participants. To compensate for drop-outs during the study and for patients with incomplete data (= patients with incomplete reference standard test results) that have to be excluded we plan to enrol 400 patients in our study. Considering the current number of patients that are regularly referred to our outpatient clinic for screening for endocrine hypertension and considering the feasibility to perform the SIT and the other diagnostic procedures at our clinic we plan to recruit these 400 patients within 3 years.

### Statistical analysis

Receiver-operating characteristics (ROC) curves will be calculated and graphed to show the characteristics of the AARR as a screening test for PA. In addition, we will also present the ROC curve for the SIT in diagnosing PA. For patients with initial beta-blocker therapy we will graph and compare the ROC curve for the AARR before (screening test) and after (second AARR determination) discontinuation of beta-blockers. We will also show the ROC curve for the ARR and we will compare the ROC curve for the AARR and ARR in diagnosing PA. ROC curves will be compared by the method of DeLong [31]. Correlation analysis will be performed of active renin concentrations and plasma renin activity. Apart from this, we will also perform the correlation analysis of aldosterone concentrations determined by RIA and by liquid chromatography – tandem mass spectrometry and we will show the Bland-Altman plot of the aldosterone values obtained by these two methods [32]. In patients with 24 hours urine sodium concentrations above 200 mEq, we will present the sensitivity and specifity of the SIT for diagnosing PA by using 24 hours urine aldosterone levels as the reference test. Statistical analyses will be performed by Analyse-it software (Analyse-it software, Ltd., Leeds, UK) [33] and by the SPSS 16.0 statistical package (SPSS, Inc., Chicago, IL, USA). A p-value below 0.05 will be considered statistically significant.

### Ethical issues

Our study population consists of patients with a high probability of PA, thus justifying an extensive screening procedure for this disease in view of the fact that patients with PA are at excess risk of cardiovascular disease which can be significantly reduced by a targeted treatment in diseased patients [1]. The SIT is considered a safe confirmatory test and previous studies have shown no significant side effects apart from occasionally observed increases in blood pressure that could all be well controlled [1-3,21,34]. Towards this, we want to note that blood pressure and heart rate will be regularly (all 30 minutes) measured during the SIT. We plan to perform the SIT in all study participants because this test can only be accurately validated if patients with and without PA are tested [10]. However, to avoid needless SIT in a relevant number of patients without PA we will perform interim analyses after the inclusion of 100 and 200 patients. If the proportion of patients without any elevated AARR (AARR of ≥ 5.7 including an aldosterone levels ≥ 9 ng/dL) exceeds 75% after inclusion of 100 patients and/or 50% after inclusion of 200 patients, we will stop to perform the SIT in such study participants. Then we would, until the end of the GECOH study, perform the SIT only in patients with at least one elevated AARR. The GECOH study was approved by the ethics committee at the Medical University of Graz, Austria.

## Discussion

The high prevalence among hypertensive patients and the great benefit of a targeted treatment in patients suffering from PA underline the importance of screening for this disease. The results of the GECOH Study will significantly add to the currently rare data of valid prospective studies that evaluate standardised procedures for screening and diagnosing PA.

A limitation of our study is that we will include patients that are referred to a tertiary care center, thus comprising a highly selected study population, and our results may therefore not be generalizable to less selected patients or even patients in primary care settings. Another main limitation is that the reference standard test (2 determinations of the AARR plus the SIT) for the diagnosis of PA is not independent from the results of the screening test because it even incorporates the AARR of the screening test. This may be responsible for an overestimation of the accuracy of the AARR. Similarly, the accuracy of the SIT may be overestimated because it is also part of the reference standard test. Apart from this, it should be acknowledged that the current diagnostic procedures for PA are based on the consideration to show that aldosterone secretion is relatively autonomous from the renin-angiotensin system and from sodium loading. It should, however, be pointed out that the regulation of aldosterone secretion is a complex process with many influence factors that still need to be further explored in detail [35]. A main problem in this field is to differentiate whether relevant alterations of renin and/or aldosterone levels are mainly driven by dysregulations of the RAAS system itself or are secondary due to other environmental and/or genetic pathologies leading to dyshomeostasis of sodium balance [14,36]. Of particular interest are also ethnic differences regarding the regulation of sodium balance and the RAAS because previous studies suggest that patients of African ancestry might be predisposed to low renin levels [14,23,37]. We therefore do not restrict our study population to certain ethnicities but the low proportion of e.g. persons with African ancestry in Austria may limit our ability to detect ethnic differences.

The main strengths of the GECOH study are the standardised blood sampling conditions, the comparison of different laboratory methods with the parallel evaluation of the AARR and ARR in diagnosing PA and the fact that all patients are scheduled to undergo the screening and the reference standard test. Apart from this, the comparison of the SIT with the results of the 24 hours urine aldosterone measurements will further improve our knowledge about the influence of chronic salt loading on diagnostic procedures for PA.

## Competing interests

The authors declare that they have no competing interests.

## Authors' contributions

SP, AT and VS contributed to the conception and design of the study. SP and AT drafted the manuscript. All authors have revised the manuscript for important intellectual content and gave final approval of the version to be published.

## Pre-publication history

The pre-publication history for this paper can be accessed here:


